# Stakeholder views on the implementation of the UK’s Antimicrobial Resistance (AMR) National Action Plan (2019–2024) in relation to AMR in the environment

**DOI:** 10.1080/16549716.2025.2543101

**Published:** 2025-08-28

**Authors:** Holly J. Tipper, Isobel C. Stanton, Rebecca E. Glover, Agata Pacho, Nicholas Mays, Andrew C. Singer

**Affiliations:** a Molecular Ecology, UK Centre for Ecology and Hydrology, Wallingford, UK; b Department of Health Services Research & Policy, London School of Hygiene & Tropical Medicine, London, UK

**Keywords:** Antimicrobial resistance (AMR), one health, research policy, environmental health, stakeholder engagement

## Abstract

**Background:**

Antimicrobial resistance (AMR) in the environment is an important component of One Health AMR research and is increasingly incorporated into AMR National Action Plans (NAPs), including the UK’s AMR NAP ‘Tackling antimicrobial resistance 2019–2024’. However, implementation of the environmental commitments has yet to be evaluated.

**Objective:**

In this study, we aimed to understand UK stakeholder perspectives on the delivery and implementation of the environmental components of the UK’s AMR NAP 2019–2024, with a particular focus on wastewater, which could be used to inform the 2024–2029 NAP.

**Methods:**

We undertook semi-structured, qualitative interviews with informed UK stakeholders to discuss how the NAP had been implemented and future directions relevant to environmental AMR.

**Results:**

Two main themes emerged from the interviews: 1) the perception of ‘risk’, and 2) barriers that have hampered policy action. Some wanted more evidence to inform policy and mitigations, particularly concerning the relative risk posed by different pollution sources in driving and maintaining AMR in the environment, and the risk posed by transmission of AMR from the environment to humans. Where evidence was lacking, several academics and regulators proposed that policy action could be justified based on the precautionary principle.

**Conclusions:**

Although we do not know the impact environmental exposure plays in driving clinical AMR infections relative to other sources, evidence suggests that exposure to environmental and wastewater sources may play a role, and thus requires policy interventions. Government leadership is critical for ensuring the uptake of environmental AMR research to inform mitigation and interventions based on the precautionary principle.

## Paper Context

- **Main findings**: Tensions in the environmental AMR community arose regarding risk; specifically, the perceived need for more evidence and to act under the precautionary principle emerged as central in environmental AMR policy. Technical barriers, such as the lack of agreed surveillance markers, and no clear targets or baselines, combined with funding constraints, have hampered policy progress.
- **Added knowledge**: This study provides knowledge on what stakeholders deem important to progress in the field of environmental AMR research, as well as regulation and mitigatory action in this area, with a particular focus on wastewater.
- **Global health impact for policy and action**: These findings highlight the need for ensuring that the environment is incorporated as a key part of global action plans. It also highlights the requirements of key stakeholders involved in the research, regulation or implementation of environmental AMR commitments.

## Background

Antimicrobial resistance (AMR) is a public health threat, with 1.27 million deaths directly attributed to bacterial AMR in 2019 [1]. AMR is considered a ‘One Health’ issue, which mobilises multiple sectors, including veterinary, public health and environmental sectors, to create long-term, sustainable solutions [2,3]. In environmental settings, AMR microorganisms and resistance-driving chemicals (ARDCs) can be released from anthropogenic inputs, including treated and untreated wastewater, and agricultural runoff from crop treatment and animal waste [4]. The presence of AMR in the environment poses a risk to human health through multiple exposure routes, including through recreational activities in sewage-impacted waters [5], which can potentially lead to colonisation by AMR microorganisms [6].

Internationally, AMR is recognised by the United Nations General Assembly as an urgent global health threat [7,8] and the World Health Organisation (WHO) has offered guidance to member states in developing National Action Plans (NAPs) on AMR [9,10].

In the UK, documentation on tackling AMR in the decades preceding the development of formalised NAPs, including the ‘UK AMR Strategy and Action Plan’ (2000) [11], and the ‘UK Five Year Antimicrobial Resistance Strategy 2013 to 2018’ [12]. The Department of Health and Social Care also published a 20-year vision for AMR [13] alongside the UK’s five-year NAP ‘Tackling antimicrobial resistance 2019–2024’ [14], with the NAP setting out the opinions and actions supporting the 20-year vision. Environmental AMR features throughout the 2019–2024 NAP in ambitions and challenges, generally to deepen understanding about AMR and ARDCs in the environment and to minimise their contamination and dissemination. Action around wastewater and AMR was not explicitly included in the nine ambitions of the 2019–2024 NAP, however it was discussed in relation to some, for example, Ambition 6: Minimise environmental spread [14].

Evaluation of the implementation of NAPs is critical to the success of future AMR policies and outcomes. Previously, studies have undertaken evaluations of global NAPs (e.g. Willemsen et al. (2022) [15] and Charani et al., (2023) [16]), and an evaluation of the UK’s AMR NAP 2013–2018 has been undertaken [17,18]. The UK’s AMR NAP 2014–2019 has been evaluated by Pacho et al. 2025 [19] and Bennani et al. 2024 [20] regarding both clinical and agricultural targets, respectively. The UK Government’s response to the AMR crisis in England has also recently been assessed by the National Audit Office [21]. In addition, Neale and Cullen (2024) evaluated the 2019–2024 NAP through roundtable discussions [22] and briefly discussed AMR in the environment, but largely focused on agriculture and food security [22]. However, although qualitative analyses of environmental commitments relating to AMR have been undertaken in other regions (e.g. investigating barriers to environmental surveillance of AMR in low- and middle-income countries [23]), no such evaluation has been undertaken regarding the environmental commitments and implementation of UK AMR NAPs. In this study, we aimed to understand UK stakeholder perspectives on the delivery and implementation of the environmental components of the UK’s AMR NAP (2019–2024). We used semi-structured interviews with UK stakeholders to discuss how the NAP had been implemented and future directions relevant to environmental AMR, particularly relating to wastewater, as wastewater is perceived as a significant contributor of AMR to the natural environment. Stakeholders were chosen for their relevance to what was perceived as a largely unaddressed challenge within a One Health NAP, similar to an approach taken by (23). This work was undertaken as part of a wider evaluation of the One Health components of the 2019–2024 NAP and was delivered to the UK Government to inform the development of the new UK’s AMR NAP 2024–2029 [24].

## Methods

### Qualitative interviews

For this study, we have included the consolidated criteria for reporting qualitative research (COREQ) checklist (Supplementary Materials 1), as described in Tong et al. (2007) [25].

#### Participants

Professionals were contacted from three sectors: government/environmental regulators (*n* = 5); wastewater industry (*n* = 6); and academia (*n* = 6). Candidates were chosen based on their relevant expertise and potential role in delivering under the NAP and were invited to interview by email (Supplementary Materials 2). Of the 17 potential participants contacted, seven either did not respond, declined or suggested alternative participants, resulting in 10 participants (Table 1). All participants were contacted before interview with a participant information sheet and were asked to sign a consent form (Supplementary Materials 3).

**Table 1.** Overview of interview participant numbers by country and sector.

Country	England	Northern Ireland	Scotland	Wales	Total
Government/Environmental Regulators	1	1	1	1	4
wastewater industry	1	0	1	1	3
Academic	1	1	1	0	3
Total	3	2	3	2	10

#### Semi-structured interviews

Topic guides were developed (Supplementary Materials 4), with questions around: 1) how the regulators and wastewater industry have addressed or are planning to address environmental challenges in the UK AMR NAP; and 2) expert judgement (e.g. what needs to be done in the future, how is the UK performing in an international context, etc.). Each interview was conducted by ICS and HJT via Microsoft Teams and lasted approximately 30–60 minutes. Interviews were conducted in January and February 2023, audio recorded on an encrypted device and sent for transcription.

### Data analysis

Interview transcripts were analysed to identify common themes. A coding framework (Supplementary Materials 5) was developed, and transcripts were coded by hand with data compiled into themes using Microsoft Excel. ICS and HJT identified themes, undertook coding, and analysed data, in consultation with AP and RG. Given the paucity of actors in these sectors, due attention was given to ensuring anonymity. Participants were anonymised in this and other subsequent reporting by excluding identifiable information (e.g. UK country) and instead are referred to by their broad sector.

### Ethical approval

This study was reviewed and approved by the Health Research Authority (REC Ref: 22/HRA/3073) and the London School of Hygiene and Tropical Medicine Ethics Committee (LSHTM Ethics Ref: 27930).

## Results

Two main themes emerged from the interviews: conversations around risk, and barriers to action.

### Risk

Participants conceptualised risk in several ways: the relative importance of environmental AMR within the One Health agenda, the risk of investments not yielding sufficient returns, and as the risk of inaction.

#### Perceptions of relative risk among one Health sectors

Overall, participants discussed different conceptualisations of ‘risk’ and how it drives monitoring or funding. One regulator stated:

Risk is about scale or magnitude of […] harm […] and the probability of that happening, and who are we interested in as receptors […]. Mainly, I think it’s humans but to some degree it will be animal, particularly valued animals, so livestock […]. And […] the environment […] I think human is where the main drive’s going to be. (Environmental regulator 1)

An academic further described a risk hierarchy; it was human health and not simply the ‘risk to the environment’ that is considered important to communicate to policymakers, as:

All the effort of the research community in this area is trying to understand the role of the natural environment […] in the emergence and transmission of [AMR] infections in humans. (Academic 1)

The concept of relative risk was discussed by participants in reference to the risk posed by different pollution sources and the risk of different exposure routes to human health. Specifically, the relative risk from wastewater or combined sewer overflows (CSOs) in comparison to other sources. One wastewater industry representative suggested that the benefits of mitigation measures to reduce AMR pollution from wastewater treatment works need to be shown before the wastewater industry is likely to invest in improved treatment:

[…] if you did nothing with the prescribing in the human population and you spent billions cleaning up wastewater, what impact would that have on overall antimicrobial resistance? […] there’s a lot of knowledge about the existence of antimicrobials and […] [AMR] but come to the mitigations, I don’t think there’s the same evidence base […] its fine stating ‘we’ve reduced all the antimicrobials coming out of our wastewater treatment works.’ Has that made a difference? Are you just spending a big clump of money doing it, but has it made a difference? [We need to] go with the [action] that will deliver the biggest benefit [and] not which one is the easiest to do. (Wastewater industry 1)

A second wastewater industry representative framed the problem similarly:

[…] it’s understanding, well what are the risks? And should we be looking a lot more at the source control approach? How do you reduce the prescribing of antibiotics? And for that matter, use of antibiotics in agriculture as well. (Wastewater industry 2)

Similarly, a different wastewater industry participant related the cost to the sector of resolving a problem of unknown size (and severity):

What I’m always wary of is someone saying […] ‘it’s a privatised water industry, they’ll pick up the tab for doing something’ and we could spend billions of pounds addressing one percent of the national problem. But if it’s billions of pounds and it’s 30% of the problem, then it’s a different equation entirely. So that’s my one plea, is who looks at what the risk in the round? Where are the optimal interventions? (Wastewater industry 2)

The industry framing around the scale of the problem was predicted by environmental regulators and academics alike:

I think if you ask the water companies, they will say […] ‘are [we] providing the biggest source and the biggest risk?’ and ‘shouldn’t other industries or groups also be contributing [to the solutions]?’ (Environmental regulator 1).

It seems totally obvious to us as academics or stakeholders […] that […] if you stop [CSO] inputs you’re going to significantly reduce [environmental] AMR […]. (Academic 2).

The relative lack of data linking exposure to environmental AMR and negative human health outcomes was noted by multiple participants, including in this statement by an academic: ‘How [does environmental AMR contribute] to the overall burden of infection? We don’t know’ (Academic 1). A regulator also stated:

[…] eventually somebody’s going to have to bite bullets and put their hands in their pockets and fund some substantial surveillance […] to begin to understand the risk […] of being exposed in the environment compare[d] to some other risk. (Environmental regulator 1)

A wastewater industry participant reiterated this knowledge gap; however, their statements were given in the context of needing information to inform decisions around costs to implement mitigation measures. They said:

[…] I have mentioned this in various forums […], no one can tell me […] my risk of picking up an AMR infection from paddling in my local stream verses catching the train to London or going to a sporting event or concert […] What are the transmission risks, what are the obvious [transmission] routes? I know the most obvious one is [going inside] a hospital […] There are obviously hotspots about where you would need to implement AMR control measures but it’s understanding that hierarchy of where […] is it the easiest to break the chain of infection […] and what are the costs of doing that? (Wastewater industry 2)

The same wastewater industry participant highlighted knowledge gaps in the current research base, particularly in the context of the recently published UK Water Industry Research (UKWIR) Chemicals Investigation Programme (CIP) 3 report on AMR, which aimed to better understand the role of wastewater treatment works on treating and transforming AMR [26]. The participant stated:

[…] the UKWIR project [showed] that you can measure what’s coming in, and you can measure what’s coming out, but you don’t actually know the impact of what’s coming out is having. (Wastewater industry 2)

#### How much knowledge is enough for action?

There were fundamental tensions among interviewees about how much evidence would be required to support environmental AMR interventions.

Academics and regulators highlighted that there is sufficient evidence of the risks of AMR, and therefore a compelling reason to act, as ‘[there is a] need to see the translation of this work into environmental standards and regulation’ (Environmental regulator 2). Another regulator agreed, stating:

[…] Some of the NAP commitments we’re working on felt a bit like an exercise in putting this off […] – we’ll ask for some research. We’ve reached the point where we should be past that now and starting to start doing things. (Environmental regulator 1)

The same participant drew comparisons with precautionary mitigation efforts in other One Health areas. When asked about future plans after their current surveillance work is complete, they said:

I work in a research team; I’m not going to say, ‘don’t do research’, but we need to move on beyond that, […] the clinicians [and] the vets, didn’t wait until they could provide you hard proof that if you withdraw use of colistin then colistin resistance starts to decline […]. Maybe we need to be thinking the same, particularly for antifungals in agriculture. (Environmental regulator 1)

This was supported by another regulator, who expressed that now is the time to implement changes and mitigate using our existing knowledge:

I think we already know enough, […] I think we need to move into some degree of surveillance Perhaps we don’t need to measure everywhere like we do with chemicals, but we need […] a broader standard programme, a mainstream one. (Government 1)

Some industry representatives stood apart, suggesting the evidence base needed strengthening, or that even with more evidence, the environmental AMR actions needed were not clear. One wastewater industry participant said ‘[the regulators] are relatively active in this space but they’re not regulating because I don’t think we’re at that stage yet’ (Wastewater industry 1). Therefore, it appears the scale of the problem, and the nature of the solution, to some extent remain contested. Moreover, another industry representative highlighted the perceived relative priority of environmental AMR: ‘For the water industry to do it, it also needs to be important for governments and regulators, and I don’t think it is’ (Wastewater industry 3).

One wastewater industry representative even expressed there may be hidden risks to eliminating antibiotic residues:

[…] if you take something out, does that create an environment for something else to thrive that might be worse than the thing that we’ve taken out. (Wastewater industry 1)

As well as requiring more evidence to act, the water industry also discussed the financial implications of mitigating potential risks, with a particular focus on CSOs. CSOs (also referred to as sewage spills, sewage discharges, outfalls, etc., by participants) release untreated sewage into aquatic environments, potentially posing a public health risk [27]. The costs of tackling CSOs were also discussed by all wastewater industry representatives. Cost estimations from participants ranged between ‘£50-something billion’ and ‘£60–100 billion’ to meet potential targets of ≤10 discharges per year in England. Industry representatives discussed desires to see cost–benefit analyses indicating to what extent improvements in CSO discharges will affect environmental AMR and often hypothesised that spending money in other sectors may help minimise risks associated with polluting inputs and AMR:

[…] if you could divert some of the billions and billions of pounds that’s going to be spent on the water company assets, to local authorities and to the Highways Agency, you’d make a much, much better start to dealing with storm overflows and then the overflows that occur a sewerage works as well. (Wastewater industry 3)

Environmental regulators also acknowledged the costs of wastewater treatment improvements, with one participant expressing applying antimicrobial discharge limits to treated effluent at this time would be ‘technically […] and financially prohibitive’ (Environmental regulator 2), and further stating that ‘we do need to be very mindful of the cost implications and the technology implications for starting to address this issue beyond what we’re doing already’ (Environmental regulator 2). However, there is some contrast between industry and regulator/government opinions, often with the industry questioning the risk posed by CSOs, particularly in relation to the required capital investment, whereas regulators are focused on gathering the evidence to demonstrate the scale of the problem caused by CSOs and other polluting inputs.

Overall, participants had conflicting opinions of ‘risk’ and on evidence requirements prior to implementing mitigating measures to tackle pollution sources that increase environmental AMR. Wastewater industry representatives often suggested that data are needed to inform both the relative risk of different pollution sources on environmental AMR and the relative risk from the environment on clinical AMR, before mitigation measures can be implemented. In addition, some called for cost–benefit analyses. In contrast, many non-industry participants thought there was sufficient compelling evidence to act now.

### Barriers to policy action

#### Technical barriers: standardisation

Participants described barriers to UK environmental regulation including the lack of consensus around surveillance markers and baseline measurements, funding constraints and the de-prioritisation of the environment in policy. Some participants hesitated to begin monitoring due to the absence of standardised approaches. They cited the need for a new surveillance method or lack of agreement in current methods. Additionally, there was limited consensus on appropriate surveillance markers (e.g. specific genes or organisms of interest). One participant stated:

[…] we need a proper surveillance design and we’re not there yet because there are so many different ways of measuring [AMR] […] we probably need a new method, […] If you take another few years to say, ‘OK, this is the method,’ then already time has passed so I think we should start […] some kind of surveillance. (Government 1)

Another participant agreed that monitoring will require a different approach to other types of surveillance:

There [is] a real lack of understanding of how environmental data would need to be reported; […] you can’t just necessarily use the same measures that you used for clinical or veterinary. (Academic 2)

The same participant further expressed the lack of consistency in the literature and the nuances in choosing the right surveillance marker(s):

If you’re looking at the literature there’s no consistency in how people have carried out studies. […] There’s […] such a bias to the organism or the gene of interest […] you’ve got to be able to link it to [human] health. (Academic 2)

This was echoed by a regulator, who stated ‘we don’t have the robust standards to use in licensing emissions to the environment to safeguard against AMR’ (Environmental regulator 2). The same participant offered examples of questions that need still answering, including:

[…] how do we address AMR in terms of water pollution or […] environmental pollution control? What constitutes harm? What criteria should we be using to assess harm in the environment? What standards should we be working towards in seeking to implement licence conditions and interventions? And at the moment this type of information […] is not available to us. (Environmental regulator 2)

Therefore, despite the regulators and academics largely supporting the implementation of a precautionary approach to mitigation, there was still uncertainty on the best measure of improvement.

Some participants also thought that understanding/collecting more baseline data and defining the state of AMR in the environment is still needed to inform targeted mitigation and evaluate whether it worked. For example, one academic stated ‘Right now we kind of need to know what’s out there and where it’s bad’ (Academic 2).

An environmental regulator discussed that a similar approach to the Water Framework Directive (WFD) (which characterises rivers and ranks them by their ecological and chemical health) would be useful. For example, an approach beginning with the ‘characterisation of pressures, the assessment of state, the evaluation of impact and the design of interventions, programmes of measures’ (Environmental regulator 2). The same participant also referred to the ‘DPSIR’ (Driver-Pressure-State-Impact-Response) framework [28] and expressed the need for establishing the ‘state’ part. Further, an academic and a regulator both discussed the current drive for designated inland bathing [29], with the regulator suggesting that routine testing of inland bathing waters could include both AMR and ARDCs. The academic suggested that bathing in inland waters poses a greater risk to human health in comparison to coastal bathing waters as there will be less dilution and that inland bathing designation should lead to increased investment, improved treatment, and a reduction in CSO discharges.

#### Financial barriers: research/governmental funding constraints

Financial constraints were discussed widely. The ability of regulators to only work within the confines of their core duties was mentioned by two participants, with one regulator stating ‘we do what we’re funded for at present. So, if surveillance for AMR became a requirement, then that would need paying for’ (Environmental regulator 1). This was also echoed by another regulator:

Where is the policy and the regulatory drive to do something about this; where is the funding to support that regulation? Particularly in these days of constrained resources, we need to be acutely aware of what our core work is. (Environmental regulator 2)

Constraints in funding were also mentioned by academics, particularly in regard to whether academics should fill the gaps in larger-scale environmental monitoring of AMR:

[…] perhaps it should be fulfilled by the regulators actually, but funded properly. I think […] it’s very rare to get the kind of money that you need to do that kind of study [in academia]. (Academic 2)

However, this same participant described that although regulators might be better placed to do this work, they ‘get the impression that the regulators don’t have the funding. So, they’re reliant on [academics] to do this, and we may or may not be able to fund it’ (Academic 2).

Participants linked funding to research priorities, with research areas of highest priority likely being assigned more funding. One environmental regulator explained that it had taken a long time to get funding for the Pathogen Surveillance in Agriculture, Food and Environment (PATH-SAFE) Programme [30], as it was not seen as a priority. The PATH-SAFE Programme is a UK cross-government programme, including the Environment Agency, which undertook multiple studies on environmental AMR. The environmental regulator stated:

PATH-SAFE […] came about after three abortive attempts to get that funding through the spending review mechanism […] failing every time, because it’s hard to get it up the agenda high enough. Even within an AMR context it’s hard because the clinicians will be there first. (Environmental regulator 1)

Other participants, particularly government/environmental regulator representatives, felt the environment has often been seen as a minor piece of the One Health AMR picture:

I think public health is the big one, animal health is the middle one and we’re the little one […]. (Government 1)

Human health still takes precedence, and understandably so, but there has been good collaboration between human health and animal health. I think probably the environment is further down in the pecking order. (Environmental regulator 2)

The environment always seems to end up as the poor relation. (Environmental regulator 1)

Some responses suggested that participants thought the sentiment expressed in the last quote was changing: ‘people are […] increasingly recognising the environment has an important role to play.’ (Environmental regulator 2), and:

I’m going to be talking to […] people that are involved in coordinating the National Action Plan [but] we wouldn’t have even been let through the door 10 years ago because they would have considered [the environment] to be irrelevant. If you think about it in those terms, the amount of progress is remarkable really. (Academic 1)

Overall, it was evident some representatives felt there was a general lack of funding for environmental AMR research and a de-prioritisation of the topic in a political context. However, it was also felt that some progress has been made and some were hopeful that this will improve further.

### Next steps and desirable policy outcomes

When participants were asked to describe what policy interventions should come next, they wanted to better build on existing research (e.g. research completed under the PATH-SAFE programme), improve and inform environmental surveillance, and improvements and mitigation within the wastewater industry.

#### Building on existing research

Academics, environmental regulators, and industry representatives valued different ways forward, but an academic and two environmental regulators described the importance of PATH-SAFE and the levels of recent governmental investment in environmental AMR research.

The key thing will [be] what happens when PATH-SAFE finishes, whether it transitions into embedding some of that understanding and practice that’s been scoped in PATH-SAFE into their routine activity. (Academic 1)

An environmental regulator explained that: ‘the first thing we need to do is digest what comes out of PATH-SAFE and [decide] if we are [going] to do anything more’, for which ‘the agency will require more funding’ (Environmental regulator 1). Another regulator mentioned financing, however, stating that: ‘there is no shortage of investment in terms of research at the moment, and […] I’m keen to see […] the translation of that research into [policy] action.’ (Environmental regulator 2). Furthermore, some had concerns about whether the completion of the research done under the PATH-SAFE programme would lead to shored-up, longer-term, funding for the sector.

#### Environmental surveillance of AMR

Regardless of whether they thought more research was needed prior to action, some academics and environmental regulators called for surveillance programmes. One participant suggested that regulators should invest in a surveillance programme that:

[…] includes human, animal and environment and potentially wastewater which is essentially part of understanding AMR in human populations. (Academic 1)

Another academic stated:

I think a programme of monitoring across water bodies […] would be really helpful. Obviously, that’s a huge financial burden. So, using a sentinel species for isolate-based work and then some selected genes for genes-based work, [is] what needs to be done […] you’ve got to be able to link it to health, [and] the isolate work has some potential to actually do that. (Academic 2)

When asked about statutory monitoring of environmental AMR, a wastewater industry representative expressed that:

[…] I’d like to see the monitoring approaches refined so that they’re a lot simpler and cheaper […] you need to make it really inexpensive at a bulk level, […] the effort needs to go into making the process of analysis much, much cheaper first, and then you make your business case for a wider scale application. (Wastewater industry 3)

Some participants discussed uncertainties brought on by Brexit concerning legislative changes in Europe and how they might be transposed into UK legislation. This was in relation to the revision of the Urban Waste Water Treatment Directive (UWWTD) (91/271/EEC) [31]. The proposed revisions will include monitoring AMR in wastewater treatment works with a population equivalent >100,000 [31]. One participant expressed uncertainties about how these amendments will affect the UK water industry:

There’s a revision of the Urban Wastewater Directive […] within the European context. I certainly know the Scottish Government has said they’re going to align with EU standards for political reasons […] and the UK Government is probably not aligning for political reasons as well. (Wastewater industry 1)

Better quality surveillance was called for by stakeholders from all sectors as a priority future area; as the quotations above demonstrate, the discussion centred around how to undertake better surveillance, not its relative importance.

#### Wastewater industry action

Some academic and environmental regulator/government representatives called for improvements to wastewater treatment processes, with one participant thinking ‘[waste]water input is probably the most significant [driver]’ (Academic 2), and another stating ‘the water industry [are] the solution’ (Academic 3). Another participant stated we need to:

Improve the issues with the CSOs, which is complex, expensive and [need] to be long-term, [and] improving […] sludge management in the water industry. (Government 1)

Another regulator/government participant also called for better wastewater treatment:

[…] from my own perspective, it would be great to see better treatment of wastewater […] to stop antibiotics getting out there to limit the amount of resistant bacteria [developing]. (Environmental regulator 3)

However, when asked about their opinions of what action the wastewater industry should take, one wastewater industry representative stated:

I imagine that we would be looking [at] a greater number of […] inland bathing river designations, which […] will drive more work on disinfection of effluent into areas that people are swimming in. […] The other thing which will have some impact over the next 25 years is the government’s storm overflow reduction plan. […] That will remove one route of entry of AMR organisms into the environment. It’s a 25-year plan and [will] take some time to implement. (Wastewater industry 2)

The same participant went on to discuss the potential future of wastewater industry research into sludge amendment to land [32]:

[…] in terms of dissemination of AMR to back into the agricultural areas, we expect […] further upgrades [are] needed to meet some changes in sludge use in agriculture regulations […]. It’s not inconceivable that in the longer term, the use of biosolids in agriculture will start to look very, very different indeed. [For example,] pyrolysis or wet oxidation […] and ending up with […] a nicely sterile product in much smaller volumes. (Wastewater industry 2)

Another wastewater industry participant stated the overall improvements that need to be made with respect to wastewater, CSOs and AMR:

The work that the industry needs to do is [to] continually [improve] the level of treatment and […] transportation of the wastewater to the treatment [plant] so that we’re minimising the untreated sewage that’s going into the environment. (Wastewater industry 1)

Overall, it was felt by some that the wastewater industry had a significant role to play in mitigating environmental AMR. The prioritisation of any improvements, however, will likely vary according to the regulation – which in turn will depend on and reflect political priorities, emerging or escalating evidence and stakeholder interest.

## Discussion

This study sought stakeholder views on the implementation of the UK’s AMR NAP 2019–2024 [14] focusing on environmental AMR challenges. Interviews with UK academics, government, regulators and wastewater industry representatives highlighted two core themes: 1) the perception of ‘risk’ and the need for more evidence and 2) barriers hindering policy action.

A common concern was the need for more evidence in areas of significant uncertainty. Environmental regulators and the wastewater industry highlighted gaps in data quantifying AMR risks from wastewater compared to other pollution sources and the potential risks to human health. Although gaps exist in the academic literature, research is increasingly addressing these issues. Evidence shows that wastewater impacts environmental AMR globally [33–38]. In the UK, studies have highlighted that wastewater treatment work effluent can elevate levels of AMR in downstream sediments and biofilms [39–41]. Similarly, research has shown that antibiotic concentrations in environmental matrices can be driven by wastewater effluent [42,43] and may be high enough to select for resistance [44]. Further, the water industry has conducted its own research into this through its completed CIP 3, where AMR was investigated across the treatment processes [26], and in its ongoing CIP4 AMR work [32]. Whilst these studies show the levels of AMR, and in some cases, the direct impact of wastewater pollution, they do not investigate the relative risk of wastewater compared to other pollution sources. Providing this kind of evidence and deciphering source apportionment can be difficult, as many factors can drive environmental AMR [45]; however, catchment-based modelling approaches could be used to investigate this (e.g., [46] and [47]).

Many interviews included discussion on CSOs acting as a potential contributor to AMR spread [27]. As treated wastewater effluent can increase AMR in the downstream river environment [39], raw wastewater discharge from CSOs may further exacerbate the issue. While CSOs were discussed across all sectors, one wastewater industry participant questioned their role in AMR pollution. Limited UK data exists on AMR risks from CSOs [27], but international studies suggest that AMR increases during wet weather (when CSOs are likely to discharge) [48,49], and that CSO discharges have higher levels of AMR compared to treated effluent [50]. The recent installation of event duration monitors on storm overflows [51] has highlighted the number and duration of spills in England and Wales [52,53]. At the time of the interviews, no publicly available data were available for Scotland or Northern Ireland, making it impossible to know the extent of these at this time. A water industry participant also pointed to other contributors, such as local authorities, Highways Agency, and private drainage systems, as factors in increased flows and CSO discharges. Future research and policy should involve these stakeholders to improve evidence collection and develop effective mitigation strategies.

The wastewater industry highlighted the need for evidence on the human health risks posed by environmental AMR, particularly in relation to wastewater. One participant noted the lack of clarity on the relative risk of obtaining an AMR infection from environmental exposure compared to other sources. While research in this area is limited, it is expanding. For example, globally significant clinical resistance genes have been traced to environmental origins [54,55]. In addition, a systematic map [5] identified 40 studies linking environmental AMR to negative human health outcomes, with water environments being the most studied (e.g., [6,55–59]). Further, more recent findings on this topic are mixed, with some studies suggesting increased exposure to the environmental AMR does [60] or does not [61,62] increase risk to human health. This gap was documented in a recent Royal Academy of Engineering report commissioned by the UK’s Chief Medical Officer, titled ‘Testing the waters: Priorities for mitigating health risks from wastewater pollution’ [63].

Expanding the evidence base on the relative role different pollution sources play in influencing environmental AMR, and quantifying the relative risk to humans from exposure to environmental AMR from different pollution sources, is critical. However, it is also vital to ensure that current academic evidence is effectively communicated and shared with stakeholders to enable those responsible actors to guide mitigations where evidence shows they are required. The difficulty that academic microbiologists have with engaging with non-experts in policy was highlighted in a recent opinion piece [64].

The barriers associated with clinical AMR are well-explored in global [9] and regional AMR NAPs, while the environment is a minor component, including in the UK’s [14]. This de-prioritisation of the environment was highlighted in interviews. Unlike clinical challenges in the UK’s NAP, which often had targeted objectives, environmental objectives focused largely on building knowledge and improving the evidence base. Further, environmentally focused objectives aiming to minimise AMR and antimicrobial pollution lacked specificity. This has potentially delayed the establishment of environmental targets for monitoring and regulation, similar to findings in a previous evaluation of the UK AMR NAP 2019–2024 [22]. However, as noted by some participants, there is increasing recognition of the environment as an important component of the One Health agenda. Examples include: the progression from the UK’s 2013–2018 AMR strategy, where environment was not explicitly included in key areas for future action, to the environmentally focused ambitions within the 2019–2024 NAP, and the development of specific pilot monitoring plans (e.g. through PATH-SAFE [65]).

Some participants noted that action from the water industry will likely follow regulation from environmental regulators. It was also apparent that regulators are looking to academia for guidance on surveillance methods and regulatory targets. An AMR monitoring system needs to be affordable, easy to implement, reproducible, and fit for purpose. The choice of surveillance approach largely depends on the questions being asked and varies by location and policy objectives [66]. Therefore, it is unresolved as to what a successful AMR monitoring system should look like, i.e. what approach should be taken, what should be monitored, and what the targets and comparative baselines should be. Participants felt that a lack of consensus on surveillance markers may be causing delays, restricting policymakers to evidence-gathering. Whilst there are markers routinely used in research or suggested by academic reviews (e.g. *intI1* and *blaCTX-M* genes) [40,66–72] that could be used in pilot or large-scale surveillance programmes, none of these have been used in a national AMR monitoring system to date. There is also a need to develop a ‘target baseline’, representing the goal for any mitigation effort, which should be achievable but also poses a low risk to humans, animals and the environment. Some participants thought baseline data for environmental AMR were lacking, without which, the success of mitigation cannot be measured.

The current ambiguity around environmental AMR surveillance has the potential to continue to reinforce policy inaction. However, the World Health Organization’s Quadripartite Technical Group on Antimicrobial Resistance and Use Integrated Surveillance [73], could provide a pragmatic way forward in harmonising environmental AMR surveillance globally by developing guidance. Further, questions around whether the UK would adopt any of the recent amendments to EU legislation in the UWWTD [31] were also raised. Aligning with EU commitments around AMR surveillance in wastewater could further help to harmonise surveillance efforts. Undertaking pilot monitoring campaigns that are intensive will enable identification of surveillance markers of interest for long-term monitoring and will help to establish baselines and inform target baselines. This could include integrating environmental AMR surveillance into existing campaigns (e.g. bathing water quality monitoring), which will allow for baseline data to be generated whilst tailored monitoring is designed.

## Conclusion

Whilst we do not know the relative impact environmental exposure plays in driving clinical AMR infections, evidence suggests that exposure to environmental and wastewater sources may play a role, and thus require policy interventions. Government leadership is critical for ensuring environmental AMR can continue to secure funding to address the knowledge gaps. This ensures mitigations are evidence-based, and policy can shift from a reliance on the precautionary principle to policy founded on robust evidence.

## Supplementary Material

Supplementary_Materials_clean_ZGHA-2025-0089.R1.docx

COREQ checklist.docx

## Data Availability

The participants of this study did not give written consent for their data to be shared publicly, so due to the sensitive nature of the research, supporting data is not available.

## References

[1] Murray CJL, Ikuta KS, Sharara F, et al. Global burden of bacterial antimicrobial resistance in 2019: a systematic analysis. Lancet. 2022;399:629–12. 35065702 10.1016/S0140-6736(21)02724-0 PMC8841637

[2] World Health Organization. One Health 2025. 2025. Available from: https://www.who.int/health-topics/one-health#tab=tab 1

[3] Tasker A, Jones-Parr C, Richardson-Gool TS. Through the kaleidoscope: antimicrobial resistance, conflict, and security: new facets and frontiers of biosecurity. Wilton Park. 2025.

[4] Singer AC, Shaw H, Rhodes V, et al. Review of antimicrobial resistance in the environment and its relevance to environmental regulators. Front Microbiol. 2016;7:1728. 27847505 10.3389/fmicb.2016.01728 PMC5088501

[5] Stanton IC, Bethel A, Leonard AFC, et al. Existing evidence on antibiotic resistance exposure and transmission to humans from the environment: a systematic map. Environ Evid. 2022;11:8. 35308196 10.1186/s13750-022-00262-2 PMC8917330

[6] Leonard AFC, Zhang L, Balfour AJ, et al. Exposure to and colonisation by antibiotic-resistant E. coli in UK coastal water users: environmental surveillance, exposure assessment, and epidemiological study (beach bum survey). Environ Int. 2018;114:326–333. 29343413 10.1016/j.envint.2017.11.003

[7] United Nations General Assembly. Political declaration of the high-level meeting on antimicrobial resistance. 2024.

[8] United Nations General Assembly. Un General Assembly high-level meeting on antimicrobial resistance 2024. 2024.

[9] World Health Organization. Global action plan on antimicrobial resistance. 2016.

[10] World Health Organization. Library of AMR national action plans. 2024. Available from: https://www.who.int/teams/surveillance-prevention-control-AMR/national-action-plan-monitoring-evaluation/library-of-national-action-plans

[11] Department of Health. Uk antimicrobial resistance strategy and action plan. 2000.

[12] Department of Health and Social Care. UK 5 year antimicrobial resistance strategy 2013 to 2018. 2013.

[13] Department of Health and Social Care. UK 20-year vision for antimicrobial resistance. 2019.

[14] Department of Health and Social Care. UK 5-year action plan for antimicrobial resistance 2019 to 2024. 2019.

[15] Willemsen A, Reid S, Assefa Y. A review of national action plans on antimicrobial resistance: strengths and weaknesses. Antimicrob Resist Infect Control. 2022;11:90. 35739564 10.1186/s13756-022-01130-x PMC9229779

[16] Charani E, Mendelson M, Pallett SJC, et al. An analysis of existing national action plans for antimicrobial resistance-gaps and opportunities in strategies optimising antibiotic use in human populations. Lancet Glob Health. 2023;11:e466–e474. 36739875 10.1016/S2214-109X(23)00019-0

[17] Eastmure E, Fraser A, Al-Haboubi M, et al. National and local implementation of the UK antimicrobial resistance (AMR) strategy, 2013–2018. 2019.

[18] Glover RE, Mays NB, Fraser A. Do you see the problem? Visualising a generalised ‘complex local system’ of antibiotic prescribing across the United Kingdom using qualitative interview data. Crit Public Health. 2023;33:459–471. 38013783 10.1080/09581596.2023.2210743 PMC10388844

[19] Pacho A, Mays N, Glover RE. The appropriateness of self-care policy for urinary tract infections among women from racialised minorities and low-income households in the United Kingdom: a qualitative study. J Health Serv Res Policy. 2025;30:189–197. 39813400 10.1177/13558196251313736

[20] Bennani H, Whatford L, Myers J, et al. Progress and challenges: implementation of the UK antimicrobial resistance national action plan 2019–2024 within the beef cattle sub-sector. Antibiotics (Basel). 2024;13:839. 39335012 10.3390/antibiotics13090839 PMC11428892

[21] National Audit Office. Investigation into how government is addressing antimicrobial resistance. 2025.

[22] Neale D, Cullen L. Evaluating the national action plan (NAP) on antimicrobial resistance, and recommendations for the next 5-year NAP: a roundtable discussion. Sustain Microbiol. 2024;1:1–8. 10.1093/sumbio/qvad001 PMC13375327 42576882

[23] Peters AC, Larsson DGJ, Laxminarayan R, et al. Barriers and pathways to environmental surveillance of antibiotic resistance in middle- and low-income settings: a qualitative exploratory key expert study. Glob Health Action. 2024;17:2343318. 38813982 10.1080/16549716.2024.2343318 PMC11141306

[24] Department of Health and Social Care. The Scottish government, Welsh government, Department for Environment Food & Rural Affairs. Department of Health (Northern Ireland), Department of Agriculture EARA. Confronting antimicrobial resistance 2024 to 2029. 2024.

[25] Tong A, Sainsbury P, Craig J. Consolidated criteria for reporting qualitative research (COREQ): a 32-item checklist for interviews and focus groups. Int J Qual Health Care. 2007;19:349–357. 17872937 10.1093/intqhc/mzm042

[26] Read D, Tipper T, Newbold L, et al. The national chemical investigations programme 2020–2022 volume 1 - investigations into changes to antimicrobial resistance through wastewater and sludge treatment processes. 2023.

[27] Tipper HJ, Stanton IC, Payne RA, et al. Do storm overflows influence AMR in the environment and is this relevant to human health? A UK perspective on a global issue. Water Res. 2024;260:121952. 38906083 10.1016/j.watres.2024.121952

[28] Food and Agriculture Organization of the United Nations. Driver-pressure-state-impact-response framework (DPSIR). 2023. Available from: https://www.fao.org/land-water/land/land-governance/land-resources-planning-toolbox/category/details/en/c/1026561/

[29] Department for Environment Food & Rural Affairs, Moore R. Record number of new bathing sites get the go ahead. 2024. Available from: https://www.gov.uk/government/news/record-number-of-new-bathing-sites-get-the-go-ahead#:~:text=Following%20a%20public%20consultation%2C%2027,the%20highest%20number%20to%20date

[30] Scholtus E, Parr E. Path-safe: tracking foodborne pathogens and antimicrobial-resistant microbes: food Standards Agency. 2021. Available from: https://food.blog.gov.uk/2021/11/23/path-safe-tracking-foodborne-pathogens-and-antimicrobial-resistant-microbes/

[31] Directive of the european parliament and of the council concerning urban wastewater treatment (recast). 2024.

[32] UK Water Industry Research. Introducing the 4th phase of the chemical investigations programme - Jenni Hughes, UKWIR strategic programme manager. 2024. Available from: https://ukwir.org/introducing-the-4th-phase-of-the-chemical-investigations-programme-jenni-hughes-ukwir-strategic-programme-manager

[33] Mukherjee M, Laird E, Gentry TJ, et al. Increased antimicrobial and multidrug resistance downstream of wastewater treatment plants in an urban watershed. Front Microbiol. 2021;12:657353. 34108949 10.3389/fmicb.2021.657353 PMC8181147

[34] Osinska A, Korzeniewska E, Harnisz M, et al. Small-scale wastewater treatment plants as a source of the dissemination of antibiotic resistance genes in the aquatic environment. J Hazard Mater. 2020;381:381. 10.1016/j.jhazmat.2019.121221 31561123

[35] Quintela-Baluja M, Abouelnaga M, Romalde J, et al. Spatial ecology of a wastewater network defines the antibiotic resistance genes in downstream receiving waters. Water Res. 2019;162:347–357. 31295654 10.1016/j.watres.2019.06.075 PMC6650630

[36] Cacace D, Fatta-Kassinos D, Manaia CM, et al. Antibiotic resistance genes in treated wastewater and in the receiving water bodies: a pan-European survey of urban settings. Water Res. 2019;162:320–330. 31288142 10.1016/j.watres.2019.06.039

[37] Bueno I, Verdugo C, Jimenez-Lopez O, et al. Role of wastewater treatment plants on environmental abundance of antimicrobial resistance genes in Chilean rivers. Int J Hyg Environ Health. 2020;223:56–64. 31722832 10.1016/j.ijheh.2019.10.006

[38] Banu RA, Alvarez JM, Reid AJ, et al. Extended spectrum beta-lactamase escherichia coli in river waters collected from two cities in Ghana, 2018–2020. Trop Med Infect Dis. 2021;6:105. 34203078 10.3390/tropicalmed6020105 PMC8293421

[39] Read DS, Gweon HS, Bowes MJ, et al. Dissemination and persistence of antimicrobial resistance (AMR) along the wastewater-river continuum. Water Res. 2024;264:122204. 39116608 10.1016/j.watres.2024.122204 PMC7617467

[40] Amos GCA, Ploumakis S, Zhang L, et al. The widespread dissemination of integrons throughout bacterial communities in a riverine system. ISME J. 2018;12:681–691. 29374269 10.1038/s41396-017-0030-8 PMC5864220

[41] Lehmann K, Bell T, Bowes MJ, et al. Trace levels of sewage effluent are sufficient to increase class 1 integron prevalence in freshwater biofilms without changing the core community. Water Res. 2016;106:163–170. 27710799 10.1016/j.watres.2016.09.035

[42] Kasprzyk-Hordern B, Dinsdale RM, Guwy AJ. The occurrence of pharmaceuticals, personal care products, endocrine disruptors and illicit drugs in surface water in South Wales, UK. Water Res. 2008;42:3498–3518. 18514758 10.1016/j.watres.2008.04.026

[43] Castrignano E, Kannan AM, Proctor K, et al. (Fluoro)quinolones and quinolone resistance genes in the aquatic environment: a river catchment perspective. Water Res. 2020;182:116015. 32622132 10.1016/j.watres.2020.116015

[44] Hayes A, May Murray L, Catherine Stanton I, et al. Predicting selection for antimicrobial resistance in UK wastewater and aquatic environments: ciprofloxacin poses a significant risk. Environ Int. 2022;169:107488. 36152362 10.1016/j.envint.2022.107488

[45] Swift BMC, Bennett M, Waller K, et al. Anthropogenic environmental drivers of antimicrobial resistance in wildlife. Sci Total Environ. 2019;649:12–20. 30170212 10.1016/j.scitotenv.2018.08.180

[46] Amos GC, Gozzard E, Carter CE, et al. Validated predictive modelling of the environmental resistome. Isme J. 2015;9:1467–1476. 25679532 10.1038/ismej.2014.237 PMC4438333

[47] Tipper HJ. Using the microbiomes of aquatic organisms to investigate the effects of pollution on environmental AMR. University of Exeter; 2021.

[48] Eramo A, Delos Reyes H, Fahrenfeld NL. Partitioning of antibiotic resistance genes and fecal indicators varies intra and inter-storm during combined sewer overflows. Front Microbiol. 2017;8:2024. 29104562 10.3389/fmicb.2017.02024 PMC5655003

[49] Lee J, Beck K, Burgmann H. Wastewater bypass is a major temporary point-source of antibiotic resistance genes and multi-resistance risk factors in a Swiss river. Water Res. 2022;208:117827. 34794019 10.1016/j.watres.2021.117827

[50] Honda R, Tachi C, Yasuda K, et al. Estimated discharge of antibiotic-resistant bacteria from combined sewer overflows of urban sewage system. NPJ Clean Water. 2020;3:15.

[51] Environment Agency, Pow R. Environment Agency publishes event duration monitoring data for 2022. 2023.

[52] Dwr Cymru Welsh Water. Combined storm overflows. 2023. Available from: https://corporate.dwrcymru.com/en/community/environment/combined-storm-overflows

[53] Hafren Dyfrdwy. Regulatory library: 2022 EDM data. Available from: https://www.hdcymru.co.uk/regulatory-library/regulatory-library/#event-duration-monitor-edm-data-2020

[54] Poirel L, Kampfer P, Nordmann P. Chromosome-encoded Ambler class A beta-lactamase of Kluyvera georgiana, a probable progenitor of a subgroup of CTX-M extended-spectrum beta-lactamases. AAC. 2002;46:4038–4040. 10.1128/AAC.46.12.4038-4040.2002 PMC132763 12435721

[55] Poirel L, Rodriguez-Martinez JM, Mammeri H, et al. Origin of plasmid-mediated quinolone resistance determinant qnra. AAC. 2005;49:3523–3525. 10.1128/AAC.49.8.3523-3525.2005 PMC1196254 16048974

[56] Leonard AF, Zhang L, Balfour AJ, et al. Human recreational exposure to antibiotic resistant bacteria in coastal bathing waters. Environ Int. 2015;82:92–100. 25832996 10.1016/j.envint.2015.02.013

[57] Laurens C, Jean-Pierre H, Licznar-Fajardo P, et al. Transmission of IMI-2 carbapenemase-producing Enterobacteriaceae from river water to human. J Glob Antimicrob Resist. 2018;15:88–92. 30279153 10.1016/j.jgar.2018.06.022

[58] Schijven JF, Blaak H, Schets FM, et al. Fate of extended-spectrum beta-lactamase-producing Escherichia coli from faecal sources in surface water and probability of human exposure through swimming. Environ Sci Technol. 2015;49:11825–11833. 26338143 10.1021/acs.est.5b01888

[59] O’Flaherty E, Solimini A, Pantanella F, et al. The potential human exposure to antibiotic resistant-Escherichia coli through recreational water. Sci Total Environ. 2019;650:786–795. 30308854 10.1016/j.scitotenv.2018.09.018

[60] Rodriguez-Molina D, Berglund F, Blaak H, et al. Carriage of ESBL-producing enterobacterales in wastewater treatment plant workers and surrounding residents - the AWARE study. Eur J Clin Microbiol Infect Dis. 2021:1–16. 10.1007/s10096-021-04387-z PMC8667530 34902088

[61] Farrell ML, Chueiri A, O’Connor L, et al. Assessing the impact of recreational water use on carriage of antimicrobial resistant organisms. Sci Total Environ. 2023;888:164201. 37196970 10.1016/j.scitotenv.2023.164201

[62] Farrell ML, Chueiri A, Maguire M, et al. Longitudinal carriage of antimicrobial resistant enterobacterales in healthy individuals in Ireland - assessing the impact of recreational water use on duration of carriage. Sci Total Environ. 2023;905:167100. 37717747 10.1016/j.scitotenv.2023.167100

[63] Royal Academy of Engineering. Testing the waters: priorities for mitigating health risks from wastewater pollution. 2024.

[64] Fady P-E. Policy in practice: pathways to maximizing microbiologists’ impact in UK policymaking. Sustai Microbiol. 2025;2:qvaf002. 10.1093/sumbio/qvaf002 PMC13375337 42576887

[65] Environment Agency. Pilot surveillance of antimicrobial resistance in river catchments in England. Bristol: Environment Agency; 2024.

[66] Hart A, Warren J, Wilkinson H, et al. Environmental surveillance of antimicrobial resistance (AMR), perspectives from a national environmental regulator in 2023. Euro Surveill. 2023;28:2200367. 36927720 10.2807/1560-7917.ES.2023.28.11.2200367 PMC10021475

[67] Berendonk TU, Manaia CM, Merlin C, et al. Tackling antibiotic resistance: the environmental framework. Nat Rev Microbiol. 2015;13:310–317. 25817583 10.1038/nrmicro3439

[68] Amos GC, Hawkey PM, Gaze WH, et al. Waste water effluent contributes to the dissemination of CTX-M-15 in the natural environment. J Antimicrob Chemother. 2014;69:1785–1791. 24797064 10.1093/jac/dku079 PMC4054988

[69] Yin X, Chen X, Jiang XT, et al. Toward a universal unit for quantification of antibiotic resistance genes in environmental samples. Environ Sci Technol. 2023;57:9713–9721. 37310875 10.1021/acs.est.3c00159

[70] Liguori K, Keenum I, Davis BC, et al. Antimicrobial resistance monitoring of water environments: a framework for standardized methods and quality control. Environ Sci Technol. 2022;56:9149–9160. 35732277 10.1021/acs.est.1c08918 PMC9261269

[71] Bengtsson-Palme J, Abramova A, Berendonk TU, et al. Towards monitoring of antimicrobial resistance in the environment: for what reasons, how to implement it, and what are the data needs? Environ Int. 2023;178:108089. 37441817 10.1016/j.envint.2023.108089

[72] Abramova A, Berendonk TU, Bengtsson-Palme J. A global baseline for qPCR-determined antimicrobial resistance gene prevalence across environments. Environ Int. 2023;178:108084. 37421899 10.1016/j.envint.2023.108084

[73] World Health Organization. The quadripartite organizations established the technical group on integrated surveillance on antimicrobial use and resistance. 2023. Available from: https://www.who.int/news/item/26-01-2023-the-quadripartite-organizations-established-the-technical-group-on-integrated-surveillance-on-antimicrobial-use-and-resistance

