# Supplementary material for: Stakeholder views on the implementation of the UK’s Antimicrobial Resistance (AMR) National Action Plan (2019–2024) in relation to AMR in the environment
Source: Glob Health Action. 2025 Aug 28;18(1):2543101. doi: 10.1080/16549716.2025.2543101 (PMC12395619; doi:10.1080/16549716.2025.2543101)
Supplement: Supplementary_Materials_clean_ZGHA-2025-0089.R1.docx [file ZGHA_A_2543101_SM7246.docx]

Stakeholder views on the implementation of the UK’s Antimicrobial Resistance (AMR) National Action Plan (2019-2024) in relation to AMR in the environment

Holly J. Tipper^1*#^, Isobel C. Stanton^1*^, Rebecca E. Glover^2^, Agata Pacho^2^, Nicholas Mays^2^ and Andrew C. Singer^1^

* contributed equally

# corresponding author - [holtip@ceh.ac.uk](mailto:holtip@ceh.ac.uk)

1. UK Centre for Ecology and Hydrology, Wallingford, OX10 8BB

2. Department of Health Services Research & Policy, London School of Hygiene & Tropical Medicine, London, WC1H 9SH

Holly J. Tipper [holtip@ceh.ac.uk](mailto:holtip@ceh.ac.uk)

Isobel C. Stanton [isosta@ceh.ac.uk](mailto:isosta@ceh.ac.uk)

Rebecca Glover [rebecca.glover@lshtm.ac.uk](mailto:rebecca.glover@lshtm.ac.uk)

Agata Pacho [agata.pacho@lshtm.ac.uk](mailto:agata.pacho@lshtm.ac.uk)

Nicholas Mays [nicholas.mays@lshtm.ac.uk](mailto:nicholas.mays@lshtm.ac.uk)

Andrew C. Singer [acsi@ceh.ac.uk](mailto:acsi@ceh.ac.uk)

Corresponding author

Holly J. Tipper

UK Centre for Ecology and Hydrology

Wallingford

Oxfordshire

OX10 8BB

[holtip@ceh.ac.uk](mailto:holtip@ceh.ac.uk)

**Supplementary Materials 1: Consolidated criteria for reporting qualitative studies (COREQ): 32-item checklist (following Tong et al. (2007) (1))**

| **No. Item** | **Guide questions/description** | **Section reported in (page no.) and details, if required** |
| --- | --- | --- |
| **Domain 1: Research team and reﬂexivity** | | |
| *Personal Characteristics* | | |
| 1. Interviewer/ facilitator | Which author/s conducted the interview or focus group? | Section: Qualitative interviews – Semi-structured interviews (Pg. 5) |
| 2. Credentials | What were the researcher’s credentials? E.g., PhD, MD | Supplementary Materials 6: Interviewer biographies (Pg. 18) |
| 3. Occupation | What was their occupation at the time of the study? | Supplementary Materials 6: Interviewer biographies (Pg. 18) |
| 4. Gender | Was the researcher male or female? | Supplementary Materials 6: Interviewer biographies (Pg. 18) |
| 5. Experience and training | What experience or training did the researcher have? | Supplementary Materials 6: Interviewer biographies (Pg. 18) |
| *Relationship with participants* | | |
| 6. Relationship established | Was a relationship established prior to study commencement? | Yes. Five of the participants had had previous academic interactions with the research team. |
| 7. Participant knowledge of the interviewer | What did the participants know about the researcher? E.g., personal goals, reasons for doing the research | Section: Qualitative interviews – Participants (Pg. 5), and in Supplementary Materials 2: Email to potential interview participants (Pg. 6-7) and Supplementary Materials 3: Participant information sheet and consent form (Pg. 8-11).  Participants were briefed on the purpose of the study and its objectives over email before consenting and also verbally before interviews began. The interviewers obtained signed consent from the interviewees before proceeding. |
| 8. Interviewer characteristics | What characteristics were reported about the interviewer/ facilitator? E.g., Bias, assumptions, reasons and interests in the research topic | Supplementary Materials 6: Interviewer biographies (Pg. 18) |
| **Domain 2: study design** | | |
| *Theoretical framework* | | |
| 9. Methodological orientation and Theory | What methodological orientation was stated to underpin the study? E.g., grounded theory, discourse analysis, ethnography, phenomenology, content analysis | Section: Methods (Pg. 4-6) |
| *Participant selection* | | |
| 10. Sampling | How were participants selected? E.g., purposive, convenience, consecutive, snowball | Section: Qualitative interviews – Participants (Pg. 5)  Participants were selected in a purposive manner, and snowball approach if approached participants declined. |
| 11. Method of approach | How were participants approached? E.g., face-to-face, telephone, mail, email | Section: Qualitative interviews – Participants (Pg. 5)  Participants were contacted via email. |
| 12. Sample size | How many participants were in the study? | Section: Qualitative interviews – Participants (Pg. 5)  Ten participants. |
| 13. Non-participation | How many people refused to participate or dropped out? Reasons? | Section: Qualitative interviews – Participants (Pg. 5)  A total of 17 potential participants were contacted, of which, seven either did not respond, declined or suggested alternative participants, resulting in ten participants. |
| *Setting* | | |
| 14. Setting of data collection | Where was the data collected? E.g., home, clinic, workplace | Section: Qualitative interviews – Semi-structured interviews (Pg. 5) |
| 15. Presence of non-participants | Was anyone else present besides the participants and researchers? | No |
| 16. Description of sample | What are the important characteristics of the sample? E.g., demographic data, date | Section: Qualitative interviews – Semi-structured interviews (Pg. 5-6) – Table 1. |
| *Data collection* | | |
| 17. Interview guide | Were questions, prompts, guides provided by the authors? Was it pilot tested? | Section: Qualitative interviews – Semi-structured interviews (Pg. 5)  Questions were not provided nor was the study pilot tested. |
| 18. Repeat interviews | Were repeat interviews carried out? If yes, how many? | No |
| 19. Audio/visual recording | Did the research use audio or visual recording to collect the data? | Section: Qualitative interviews – Semi-structured interviews (Pg. 5)  Audio from interviews was recorded on a encrypted device. |
| 20. Field notes | Were ﬁeld notes made during and/or after the interview or focus group? | Section: Qualitative interviews – Semi-structured interviews (Pg. 5)  No notes were made, but audio of the interview was transcribed. |
| 21. Duration | What was the duration of the interviews or focus group? | Section: Qualitative interviews – Semi-structured interviews (Pg. 5)  Interview duration ranged from 30-60 minutes. |
| 22. Data saturation | Was data saturation discussed? | Yes, informally within the research team. |
| 23. Transcripts returned | Were transcripts returned to participants for comment and/or correction? | No |
| **Domain 3: analysis and ﬁndings** | | |
| *Data analysis* | | |
| 24. Number of data coders | How many data coders coded the data? | Two data coders (HJT and ICS) |
| 25. Description of the coding tree | Did authors provide a description of the coding tree? | Supplementary Materials 5: Data coding (Pg. 17) |
| 26. Derivation of themes | Were themes identiﬁed in advance or derived from the data? | Section: Data analysis (Pg. 6) |
| 27. Software | What software, if applicable, was used to manage the data? | Section: Data analysis (Pg. 6) |
| 28. Participant checking | Did participants provide feedback on the ﬁndings? | No |
| *Reporting* | | |
| 29. Quotations presented | Were participant quotations presented to illustrate the themes/ﬁndings? Was each quotation identiﬁed? E.g., participant number | Section: Results (Pg. 6-19)  Yes. |
| 30. Data and ﬁndings consistent | Was there consistency between the data presented and the ﬁndings? | Yes |
| 31. Clarity of major themes | Were major themes clearly presented in the ﬁndings? | Yes |
| 32. Clarity of minor themes | Is there a description of diverse cases or discussion of minor themes? | Section: Discussion (Pg. 19-23)  Yes. |

**Supplementary Materials 2: Email to potential interview participants**

Below is a template for the emails sent to potential UK interview participants. Actual emails sent may have been slightly amended from the below email to personalise it to the recipient.

“Dear [name],

I am working on a Department of Health and Social Care funded project reviewing the implementation and response of the UK to “Tackling antimicrobial resistance 2019–2024: the UK Antimicrobial Resistance National Action Plan 2019- 2024.”

I am part of the team focusing on the environmental aspects of the AMR national action (AMR NAP) plan alongside Holly and Andrew (CC'd). In addition, we are working with colleagues from the London School of Hygiene and Tropical Medicine who are working on other "One Health" aspects of the AMR NAP (Lead PI: Professor Nick Mays).

As part of this work, we have written a “Review of Government, Regulator and Water Industry Activities Addressing the Environmental Components of the UK AMR National Action Plan (2019-2024)”. To complement this, we will be conducting qualitative interviews (aiming for 30-60 minutes in length) that aim to gather the opinions of representatives from academia, environmental regulators and the water industry from all four nations of the UK on how the UK has implemented/responded to the AMR NAP. We have identified you as someone who we would like to interview in relation to the response of your sector. Is this something you would be interested in?

If you are interested, we are hoping to undertake interviews in early 2023 (end of January and February). If you would not like to be interviewed or do not have time, we would be grateful if you could put us in contact with a colleague from the same UK nation who you deem relevant to participate.

The study has been reviewed and approved by the Health Research Authority and the LSHTM ethics committee.

Please do get in touch if you have any questions.

We look forward to hearing from you.

Best wishes,

Isobel”

**Supplementary Materials 3: Participant information sheet and consent form**

Below, the participant information sheet and the consent form sent to all participants can be found. These were sent prior to the interviews taking place and participants were required to read both and sign and return the consent form.


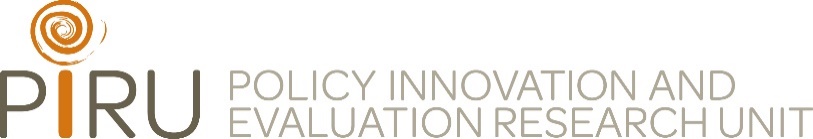

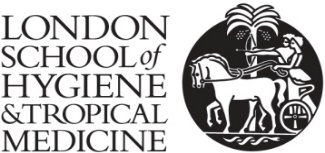


Implementation evaluation of the UK’s antimicrobial resistance five-year national action plan (NAP)

**Information form**

We are contacting you to ask you to take part in our research. Before you decide, please read this information sheet which describes the research project.

***What is the Evaluation of the UK Five Year Antimicrobial National Action Plan?***

The Policy Innovation and Evaluation Research Unit (PIRU), which is based at the London School of Hygiene and Tropical Medicine, was commissioned by the National Institute for Health Research (NIHR) to evaluate the implementation of the UK’s five-year antimicrobial resistance (AMR) National Action Plan (NAP), in order to contribute to the development and implementation of future AMR policy and assess the impact of some specific interventions in the NAP. The NAP focusses on reducing the need for, and unintentional exposure to, antimicrobials; optimising their use and investing in innovation and supply of, and access to, antimicrobials.

*Why am I being approached for an interview?*

We are interviewing people to obtain their views on a range of issues: for example, to find out how the AMR national action plan is intended to work, and what its effects might be. You have been approached for an interview because you have been involved in the design and/or implementation of the Strategy, either directly (through your role as a policymaker or guideline developer) or indirectly (through your position or organisation having some direct or indirect involvement in antibiotics, their sale, prescription, use, or byproducts or contact with the above).

*Why have I been given an information sheet and consent form?*

Before you agree to be interviewed, it is important that you understand why the research is being done and what it will involve. Your information is very important to the study and anything you tell us will be anonymised if that is your preference. Please ask the interviewer about anything that is not clear or if you would like more information, or if you would like to talk to someone about the study please contact Rebecca Glover (***redacted*** or phone ***redacted***).

*Do I have to take part?*

Taking part is voluntary and you are free to stop the interview at any time without a reason. You can also decide not to answer specific questions, without giving a reason. Interviews are confidential, and we will not discuss your opinions/views with your colleagues.

*What do I have to do?*

If you decide to take part, you will be visited by a trained interviewer at a suitable time. If this is not possible, we will conduct the interview by telephone. The interview will last about 60 minutes. During the interview we will ask you about your experiences of, and/or your views on, various aspects of the AMR National Action Plan, including topics such as diagnostics, patient pathways, the health system(s), veterinary medicine, the environment, the patient or public experience, self-care, and antibiotic prescribing more broadly, depending on your areas of expertise. We would like to record the interview with your permission, simply for reasons of accuracy. The interview will then be transcribed.

Any reports including details of the interviews will not identify the name of anyone who has requested anonymity, and interviewee details will be kept confidential. If you prefer to have quotes in our reports attributed to you, we will provide you with an opportunity to comment on the use of the quotes. If you do not agree with the use of the quotes, we will anonymise the quotes, or if necessary, withdraw the quotes. These interviews will not be remunerated.

*Who can I contact for more information?*

If you would like to take part in the research, or have any questions that you would like to ask before you decide, please contact Rebecca Glover (***redacted)***.

*Who to contact to make a complaint?*

If you have a concern about any aspect of this study, you should ask to speak to Rebecca Glover who will do her best to answer your questions (***redacted***). If you remain unhappy and wish to complain formally, you can do this by contacting Patricia Henley at ***redacted***.

**The Project Team**

The project team includes Nicholas Mays, Mustafa Al-Haboubi, Houda Bennani, Jennifer Bostock, Rebecca Glover, Barbara Haesler, Agata Pacho, Becky Knowles, Louise Whatford, Isobel Stanton, Holly Tipper, Andrew Singer and CJ Iliopoulos.

**Thank you for your help.**

*This project is funded by the National Institute for Health Research*

V1 31 May 2022


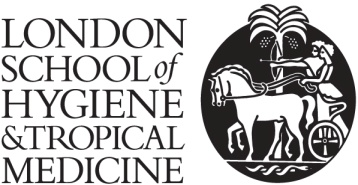

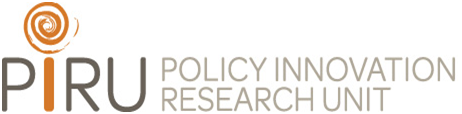


Implementation evaluation of the UK’s antimicrobial resistance five-year national action plan (NAP).

**Consent Form**

*Please initial box*

1. I confirm that I have read and understand the Information Sheet

dated 31 May 2022 (version 1) for the above study and

have had the opportunity to ask questions

1. I understand that my participation is voluntary and that I am free to withdraw

at any time without giving any reason.

1. I agree to be interviewed as part of the above study
2. I agree that the interview may be audio recorded

Name of interviewee Date Signature

Name of interviewer Date Signature

V1 31 May 2022

**Supplementary Materials 4: Topic guides**

All interviews started with the following statement:

“We’re working with colleagues from the London School of Hygiene & Tropical Medicine on evaluating the implementation of the UK’s 5-year AMR national action plan, which spans from 2019 to 24. Specifically, we are looking at how the environmental regulators of the four nations of the UK and the water industry within the UK have or are directly or indirectly addressing the environmental challenges from the UK AMR national action plan. This interview will be recorded and transcribed, which is described in more detail in the consent form. The results of interview and data collected will remain confidential.”

Environmental Regulators

1. What do you see as your day-to-day job role and how does that relate to environmental AMR?

Questions based on NAP challenges:

1. Are you aware of activities that environmental regulators are undertaking to help to understand how AMR spreads between and among humans, animals and the environment?
2. Are you aware of activities that environmental regulators are undertaking to help to minimise the spread of AMR through the environment?
3. Are you aware of activities that environmental regulators are undertaking to help to minimise antimicrobial contamination?
4. Are you aware of activities that environmental regulators are undertaking to help to develop an improved evidence base and deepening understanding on AMR in the environment?
5. In your opinion, how do you feel the water industry has responded to the environmental challenges in the national action plan?

Other questions:

1. Are you aware of any upcoming plans by environmental regulators that will help to address the previous 5 environmental challenges?
2. In your opinion, what future potential activities could environmental regulators participate in or implement to help address these issues?
3. In your opinion, how has the UK responded to the issue of environmental AMR in an international context?
4. Is there anything you wish to talk about that we haven’t covered?

Water Industry

1. What do you see as your day-to-day job role and how does that relate to environmental AMR?

Questions based on NAP challenges:

1. Are you aware of activities that water industry are undertaking to help to understand how AMR spreads between and among humans, animals and the environment?
2. Are you aware of activities that water industry are undertaking to help to minimise the spread of AMR through the environment?
3. Are you aware of activities that water industry are undertaking to help to minimise antimicrobial contamination?
4. Are you aware of activities that water industry are undertaking to help to develop an improved evidence base and deepening understanding on AMR in the environment?
5. In your opinion, how do you feel environmental regulators have responded to the environmental challenges in the national action plan?

Other questions:

1. Are you aware of any upcoming plans by water industry that will help to address the previous 5 environmental challenges?
2. In your opinion, what future potential activities could the water industry participate in or implement to help address these issues?
3. In your opinion, how has the UK water industry responded to the issue of environmental AMR in an international context?
4. Is there anything you wish to talk about that we haven’t covered?

Academia

1. What do you see as your day-to-day job role and how does that relate to environmental AMR?

Questions based on National Action Plan challenges for environmental regulators:

1. Are you aware of activities that environmental regulators are undertaking to help to understand how AMR spreads between and among humans, animals and the environment?
2. Are you aware of activities that environmental regulators are undertaking to help to minimise the spread of AMR through the environment?
3. Are you aware of activities that environmental regulators are undertaking to help to minimise antimicrobial contamination?
4. Are you aware of activities that environmental regulators are undertaking to help to develop an improved evidence base and deepen understanding on AMR in the environment?
5. Are you aware of any upcoming plans by environmental regulators that will help to address the environmental challenges from National Action Plan?
6. In your opinion, what future potential activities could environmental regulators participate in or implement to help address these issues?

Questions based on National Action Plan challenges for wastewater industry:

1. Are you aware of activities that water industry are undertaking to help to understand how AMR spreads between and among humans, animals and the environment?
2. Are you aware of activities that water industry are undertaking to help to minimise the spread of AMR through the environment?
3. Are you aware of activities that water industry are undertaking to help to minimise antimicrobial contamination?
4. Are you aware of activities that water industry are undertaking to help to develop an improved evidence base and deepening understanding on AMR in the environment?
5. Are you aware of any upcoming plans by water industries that will help to address the environmental challenges from the National Action Plan?
6. In your opinion, what future potential activities could water industry participate in or implement to help address these issues?
7. In your opinion, how has the UK responded to the issue of environmental AMR in an international context?
8. Is there anything you wish to talk about that we haven’t covered?

**Supplementary Materials 5: Data coding**

Note: The coding includes themes that were detailed in the report provided to the Department of Health and Social care but are not covered in this manuscript.

- - Communication and collaboration
    - Cross-sector
    - Cross-nation
    - Support of academic research
    - Data/knowledge sharing
    - Groups that exist (e.g., DARC, OHBP)
  - Risk
    - What is the risk? i.e., public health risk from environment
    - Relative risk of different sources to AMR and public health
      - CSO discussion - prompted
      - Privatisation
    - Precautionary principle
    - What would they like to do
  - Political context
    - Funding
    - COVID
    - Brexit
    - Potential carbon costs
    - De-prioritisation of environment
    - Likelihood to act on findings of previous research > mitigation

UK in an international context - prompted

**Supplementary Materials 6: Interviewer biographies**

Dr Holly J Tipper (PhD) is a Molecular Biologist at the UK Centre for Ecology & Hydrology, Wallingford, and was during data collection. She uses genomic approaches to understand the dynamics of antimicrobial resistance in wastewater and natural aquatic environments. She has also been part of industry- and policy-informing research in the area of environmental AMR and AMR in wastewater.

Dr Isobel C Stanton (PhD) is an Environmental Molecular Microbiologist at the UK Centre for Ecology & Hydrology, Wallingford, and was during data collection. She has used quantitative and qualitative methods to understand antimicrobial resistance in the environment and the risks to human health. She has also been part of policy-informing research in the area of environmental AMR.

**References**

1. Tong A, Sainsbury P, Craig J. Consolidated criteria for reporting qualitative research (COREQ): a 32-item checklist for interviews and focus groups. Int J Qual Health Care. 2007;19(6):349-57. Epub 20070914. doi: 10.1093/intqhc/mzm042. PubMed PMID: 17872937.
