# Supplementary material for: Stakeholder views on the implementation of the UK’s Antimicrobial Resistance (AMR) National Action Plan (2019–2024) in relation to AMR in the environment
Source: Glob Health Action. 2025 Aug 28;18(1):2543101. doi: 10.1080/16549716.2025.2543101 (PMC12395619; doi:10.1080/16549716.2025.2543101)
Supplement: COREQ checklist.docx [file ZGHA_A_2543101_SM7245.docx]

**Consolidated criteria for reporting qualitative studies (COREQ): 32-item checklist**

| **No. Item** | **Guide questions/description** | **Section reported in (page no.) and details, if required** |
| --- | --- | --- |
| **Domain 1: Research team and reﬂexivity** | | |
| *Personal Characteristics* | | |
| 1. Interviewer/ facilitator | Which author/s conducted the interview or focus group? | Section: Qualitative interviews – Semi-structured interviews (Pg. 5) |
| 2. Credentials | What were the researcher’s credentials? E.g., PhD, MD | Supplementary Materials 6: Interviewer biographies (Pg. 18) |
| 3. Occupation | What was their occupation at the time of the study? | Supplementary Materials 6: Interviewer biographies (Pg. 18) |
| 4. Gender | Was the researcher male or female? | Supplementary Materials 6: Interviewer biographies (Pg. 18) |
| 5. Experience and training | What experience or training did the researcher have? | Supplementary Materials 6: Interviewer biographies (Pg. 18) |
| *Relationship with participants* | | |
| 6. Relationship established | Was a relationship established prior to study commencement? | Yes. Five of the participants had had previous academic interactions with the research team. |
| 7. Participant knowledge of the interviewer | What did the participants know about the researcher? E.g., personal goals, reasons for doing the research | Section: Qualitative interviews – Participants (Pg. 5), and in Supplementary Materials 2: Email to potential interview participants (Pg. 6-7) and Supplementary Materials 3: Participant information sheet and consent form (Pg. 8-11).  Participants were briefed on the purpose of the study and its objectives over email before consenting and also verbally before interviews began. The interviewers obtained signed consent from the interviewees before proceeding. |
| 8. Interviewer characteristics | What characteristics were reported about the interviewer/ facilitator? E.g., Bias, assumptions, reasons and interests in the research topic | Supplementary Materials 6: Interviewer biographies (Pg. 18) |
| **Domain 2: study design** | | |
| *Theoretical framework* | | |
| 9. Methodological orientation and Theory | What methodological orientation was stated to underpin the study? E.g., grounded theory, discourse analysis, ethnography, phenomenology, content analysis | Section: Methods (Pg. 4-6) |
| *Participant selection* | | |
| 10. Sampling | How were participants selected? E.g., purposive, convenience, consecutive, snowball | Section: Qualitative interviews – Participants (Pg. 5)  Participants were selected in a purposive manner, and snowball approach if approached participants declined. |
| 11. Method of approach | How were participants approached? E.g., face-to-face, telephone, mail, email | Section: Qualitative interviews – Participants (Pg. 5)  Participants were contacted via email. |
| 12. Sample size | How many participants were in the study? | Section: Qualitative interviews – Participants (Pg. 5)  Ten participants. |
| 13. Non-participation | How many people refused to participate or dropped out? Reasons? | Section: Qualitative interviews – Participants (Pg. 5)  A total of 17 potential participants were contacted, of which, seven either did not respond, declined or suggested alternative participants, resulting in ten participants. |
| *Setting* | | |
| 14. Setting of data collection | Where was the data collected? E.g., home, clinic, workplace | Section: Qualitative interviews – Semi-structured interviews (Pg. 5) |
| 15. Presence of non-participants | Was anyone else present besides the participants and researchers? | No |
| 16. Description of sample | What are the important characteristics of the sample? E.g., demographic data, date | Section: Qualitative interviews – Semi-structured interviews (Pg. 5-6) – Table 1. |
| *Data collection* | | |
| 17. Interview guide | Were questions, prompts, guides provided by the authors? Was it pilot tested? | Section: Qualitative interviews – Semi-structured interviews (Pg. 5)  Questions were not provided nor was the study pilot tested. |
| 18. Repeat interviews | Were repeat interviews carried out? If yes, how many? | No |
| 19. Audio/visual recording | Did the research use audio or visual recording to collect the data? | Section: Qualitative interviews – Semi-structured interviews (Pg. 5)  Audio from interviews was recorded on a encrypted device. |
| 20. Field notes | Were ﬁeld notes made during and/or after the interview or focus group? | Section: Qualitative interviews – Semi-structured interviews (Pg. 5)  No notes were made, but audio of the interview was transcribed. |
| 21. Duration | What was the duration of the interviews or focus group? | Section: Qualitative interviews – Semi-structured interviews (Pg. 5)  Interview duration ranged from 30-60 minutes. |
| 22. Data saturation | Was data saturation discussed? | Yes, informally within the research team. |
| 23. Transcripts returned | Were transcripts returned to participants for comment and/or correction? | No |
| **Domain 3: analysis and ﬁndings** | | |
| *Data analysis* | | |
| 24. Number of data coders | How many data coders coded the data? | Two data coders (HJT and ICS) |
| 25. Description of the coding tree | Did authors provide a description of the coding tree? | Supplementary Materials 5: Data coding (Pg. 17) |
| 26. Derivation of themes | Were themes identiﬁed in advance or derived from the data? | Section: Data analysis (Pg. 6) |
| 27. Software | What software, if applicable, was used to manage the data? | Section: Data analysis (Pg. 6) |
| 28. Participant checking | Did participants provide feedback on the ﬁndings? | No |
| *Reporting* | | |
| 29. Quotations presented | Were participant quotations presented to illustrate the themes/ﬁndings? Was each quotation identiﬁed? E.g., participant number | Section: Results (Pg. 6-19)  Yes. |
| 30. Data and ﬁndings consistent | Was there consistency between the data presented and the ﬁndings? | Yes |
| 31. Clarity of major themes | Were major themes clearly presented in the ﬁndings? | Yes |
| 32. Clarity of minor themes | Is there a description of diverse cases or discussion of minor themes? | Section: Discussion (Pg. 19-23)  Yes. |
